# The distribution of Van Genuchten model parameters on soil-water characteristic curves in Chinese Loess Plateau and new predicting method on unsaturated permeability coefficient of loess

**DOI:** 10.1371/journal.pone.0278307

**Published:** 2023-01-04

**Authors:** Shiyue Fang, Pengfei Shen, Xinhai Qi, Fan Zhao, Yue Gu, Jiaxin Huang, Yan Li

**Affiliations:** 1 College of Geology and Environment, Xi’an University of Science and Technology, Xi’an, Shaanxi, China; 2 Gansu Shaanxi Branch of West to East Gas Transmission Company, PipeChina, Xi’an, Shaanxi, China; 3 North Shaanxi Mining Hong Liu Lin Company Limited, Yulin, Shaanxi, China; Institute of Earth and Environment, Chinese Academy of Sciences, CHINA

## Abstract

The unsaturated permeability coefficients are often used to solve geotechnical problems associated with unsaturated soils. But it is very difficult to measure. However, the unsaturated permeability coefficients can be predicted by the Soil-water Characteristic Curves (SWCCs). The Van Genuchten Model (VG model) is very rife as it’s smooth and good fitting, thus, it has the most research data. Therefore, the research data on VG model parameters (*α*, *n*, *θ*_*s*_ and *θ*_*r*_) of Malan loess in Chinese Loess Plateau are collected in the past two decades to obtain the spatial distribution characteristics of parameters. The trend surface analysis method is employed to clarify the regional scale distribution and the variation regular pattern on ArcGIS. Then the linear regression method is utilized to fit the relationship between suction and water content in three different regions of Chinese Loess Plateau, which is divided according to the properties and particle gradation. By using this relationship and the trend surface analysis contour map, the unsaturated permeability coefficient of the sample can be predicted after measuring the saturated permeability coefficient. The example verification shows that the difference between the prediction results and the experimental results is very small when the sample has the lower saturation, and the deviation is slightly larger if it has the higher saturation, but they are all within the acceptable range. This method not only saves the test cost, but also considers the physical properties of the loess in the three different regions of the Loess Plateau. With the improvement of data and the gradual improvement of sampling density, the prediction accuracy will gradually improve. It can provide convenience for solving the engineering problems of loess and water and other engineering applications.

## Introduction

The permeability coefficient, also known as the hydraulic conductivity, represents the seepage velocity of water in different media under the unit hydraulic gradient. The unsaturated permeability coefficient is related to the water content, and the value of the unsaturated permeability coefficient changes continuously with the change of the matrix suction, and the variation range can span several orders of magnitude, so it is difficult to accurately measure the unsaturated permeability coefficient. Unsaturated permeability coefficients are often used to solve geotechnical problems associated with unsaturated soils. In view of the difficulty of its measurement, many scholars have done extensive research on the prediction of unsaturated permeability coefficient. Mualem [1] made a systematic analysis of the calculation models of unsaturated soil permeability coefficient, and divided these calculation models into the following three categories: (1) empirical models; (2) statistical models; (3) macroscopic models.

Empirical models can be roughly divided into two categories. Expressing the permeability coefficient of unsaturated soils with moisture content, such as the research of Gardner [2]、Davidson et al. [3]and Campbell [4]. Or expressing the permeability coefficient of unsaturated soils with matrix suction, Richards [5], Christensen [6], Wind [7], Gardner [2], Rijtema [8], Philip [9]and Leong & Rahardjo [10]have carried out more in-depth research in this category.

The statistical models is derived from the Hagen-Poiseuille equation [11] and the Kelvin capillary model. This type of model established by the corresponding hydraulic parameters which is based on the probability of connected pores in soil under seepage [1, 12, 13]. Childs&Collis-Geroge [14] established the statistical model of unsaturated permeability coefficient in 1950, after that, Marshall [15]&Kunze [16] made improvements to this statistical model. In the process of calculating the unsaturated permeability coefficient, the soil-water characteristic curve and the volumetric moisture content test data are firstly divided into equal parts, then the water moisture is calculated based on the median value of each division, and finally apply the permeability coefficient equation to calculate the permeability coefficient.

The macroscopic models relates the permeability coefficient of unsaturated soils to the effective saturation. Such as, Gardner [2] Brooks & Corey [17] Mualem [18] have done related research, of which the Mualem model is the most representative model, which is describes the relationship between saturated permeability coefficient and soil-water characteristic curves. Because the saturated permeability coefficient is easy to measured, so the soil-water characteristic curve plays an important role in predicting unsaturated permeability coefficient. Nowadays, it has been widely used to the estimation of unsaturated soil properties.

**Fig 1:**
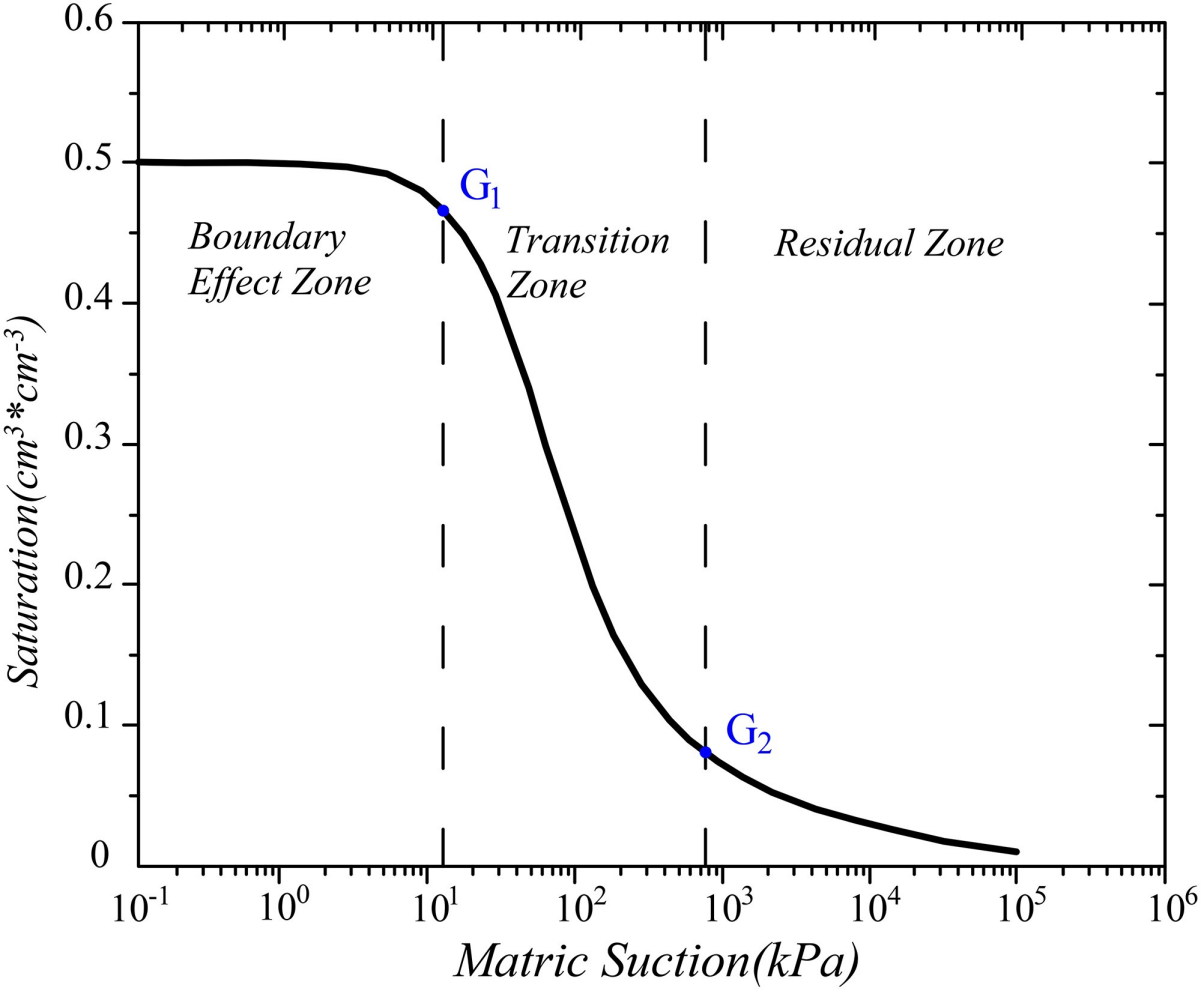
Typical soil-water characteristic curve.

The Soil–water Characteristic Curves (i.e. SWCCs) describe the relationship between water content and matrix suction. The water content can be volumetric moisture content (*θ*), gravimetric moisture content (*ω*_*W*_) or degree of saturation (*S*_*r*_). Matrix suction can also be called capillary pressure. It can be represented by (*u*_*a*_ − *u*_*w*_), where *u*_*a*_ represents pore air pressure and *u*_*w*_ represents pore water pressure [13, 19, 20]. A typical SWCC is shown in Fig 1 [21]. The SWCCs (both drying and wetting) are sigmoidal in shape, the two transition points on the drying curve are the air-entry value and residual suction value [22]. The air-entry value(G1) is defined as the matric suction at which air enters into the largest pores by drying. The residual water content (*θ*_*r*_) is the water content at which water phase is discontinuous. The suction value corresponding to *θ*_*r*_ is called the residual suction value(G2). The air-entry value and residual suction value divide the drying SWCC into three identifiable stages, namely, boundary effect zone, transition zone and residual zone [19, 23].

Generally, there are two ways to get the SWCC for a soil sample [24], direct measure methods and indirect predict methods. However, direct measure methods have some defects. Some test instrumentations are restricted by their design parameters, so, some could not measure all suction range [25], some have shortcomings of the time-consuming, special demands on the testing environment, and some need expensive testing cost [26]. So far, there are three different approaches to predict the SWCCs .1) database mining for the SWCC from similar soils, 2) prediction from the grain-size distribution(i.e. GSD) curve, 3) correlations relating parameters in the SWCC equations to soil properties [22]. Numerous studies have been performed on the last two approaches. ARYA and PARIS [27] were the first to propose a physico-empirical model for predicting the SWCC from the GSD. The GSD is translated into the pore-size distribution (i.e. PSD), which is then related to the SWCC through the capillary theory. The pore radius prediction was based on the assumption of spherical particles and cylindrical pores. The ARYA and PARIS [27] model was later modified and improved by considering the random packing of spherical particles and the influence of soil structure [28]. Recently, the PSD was directly measured and used to predict the SWCC and changes in the PSD during drying and wetting were also considered. SIMMS and YANFUL [29] developed a model for predicting the drying SWCC based on the evolution of measured PSDs for a compacted clayey soil. HU et al. [30] proposed a hysteretic SWCC model based on changes in the PSD taking account of the influence of volumetric deformation on the SWCC. There are other physico-empirical models that do not have to translate the GSD into the PSD. And The methods which relate the fitting parameters in mathematical equations to soil properties. The widely-known SWCC models, such as the Grander [2] model, BROOKS and COREY [17] model, Van Genuchten [13] model and FREDLUND & XING [31] model. Among them, the Van Genuchten [13] model is widely used, it can better fit the soil-water characteristic curves of various soils. which is:

$$\theta_{\omega =}\theta_{r}+\frac{\theta_{s}-\theta_{r}}{{\left[1+{\left(\alpha \cdot \psi \right)}^{n}\right]}^{m}}$$ (1)

Where *θ*_*ω*_ is the volumetric moisture content, *cm*^*3*^*/cm*^*3*^; *θ*_*s*_ is the saturated water content, *cm*^*3*^*/cm*^*3*^; *θ*_*r*_ is the residual water content, *cm*^*3*^*/cm*^*3*^; *ψ* is the matrix suction, *kPa*; *α* is the parameter related to the air entry value, *kPa*; *n* is the parameter controls the slope of the soil-water characteristic curve; $m=\left(1-\frac{1}{n}\right)$.

The mathematical form of the Van Genuchten Model (i.e. VG model), which accounts for an inflection point, thus it allows greater flexibility than the other model over a wider range of suction and captures the sigmoidal shape of typical curves better. Smooth transitions at the air-entry value and approaching the residual suction value condition are more effectively captured. Parameter *m* (i.e. $\left(1-\frac{1}{n}\right)$) reduces the flexibility of the VG model but significantly simplifies it. Thus resulting in greater stability during parameter optimization and permitting closed-form solution of the hydraulic conductivity function [21].

Loess soils are widely distributed in arid and semi-arid regions, covering about 10% land area of the world. Many countries in Asia, Europe, North America, South America and Africa have loess soil deposits. In China, the renowned Loess Plateau extends over 6.4×10^5^ km^2^, accounting for over 7% land area of the country [22, 32–36]. The Chinese Loess Plateau originates from aeolian dust deposit since 2.6 million years ago, which spans over the Pleistocene period (i.e. 2.4–0.01 Ma) and Holocene period (i.e. recent 0.01 Ma). Since the structure of Holocene loess and Malan loess is relatively loose, the engineering geological problems of loess are common in these two layers [34]. However, Holocene loess is usually cultivated layer, and engineering construction is mostly in Malan loess, such as retaining wall foundation, oil and gas pipeline trench foundation, etc. Therefore, the study of loess engineering properties is generally concentrated in Malan loess.

Malan loess is typically in a state of unsaturated condition. The unsaturated permeability coefficient is usually used to address geotechnical problems associated with Malan loess [37]. In order to solve engineering geotechnical problems in Malan loess better, research on the regional distribution of SWCCs, especially, VG model and the unsaturated permeability coefficient become more and more important.

In view of this, the VG model four parameters (*α*, *n*, *θ*_*s*_ and *θ*_*r*_) of Malan loess SWCCs tested by experts and scholars will be collected in Loess Plateau for recent years. The regional distribution of the VG model parameters will be elucidated by employing the trend surface analysis and the linear regression methods. And through the connection between the parameters of the VG model and the unsaturated permeability coefficient, a new method for calculating the unsaturated permeability coefficient of loess is proposed. In this way, the engineering problem related to water in loess will be solved easily and provide convenience for other engineering applications.

## Materials and methods

### Materials

SWCCs of Malan loess is the key to solve engineering problems related to loess and water. Hence, the parameters of VG model on SWCCs of Malan loess in Chinese Loess Plateau are gathered and summarized as shown in S1 Table & Table 1.

**Table 1:** VG model parameter statistics.

area	*θ*_*s*_	*θ*_*r*_	*α*	n	Data source
Xingxian	0.3900	0.1000	0.0120	--	Fan S. [38]
Jingbian	0.2740	0.0570	0.0733	1.6339	Zeng L. [39]
Shenmu	0.4550	0.0450	0.0420	1.8500	Zhao A.H. [40]
Ordos	0.4990	0.1418	0.0190	1.3565	Yang J.Y.[41]
Fuxian	0.4800	0.1700	0.0600	2.0100	Fan S. [38]
Qingyang	0.4340	0.1895	0.0276	1.5631	Li P. [22], Wang J.W. [42]
Yan’an	0.3271	0.1238	0.2002	1.3194	Nie K.Y. [43], Chen C. [44]
Liulin	0.5250	0.0368	0.0566	1.6500	Qiao P.T. [45]
Jingyang	0.4595	0.0610	0.1067	1.7005	Shi L.Y. [46], Yang H. [47]
Hequ	0.2424	0.0537	0.0860	1.9320	Jiang C.X. [48], Wang J.E. [49]
Binxian	0.5180	0.1610	0.0355	1.0200	Li P. [22]
Xining	0.4213	--	0.1920	1.2725	Bi J. [50]
Luochuan	0.5420	0.1630	0.0441	1.2800	Li P. [22]
Lanzhou	0.3414	0.0958	0.0433	1.6413	Zhang P.Y. [51], Jiang Y. [52], Liu P. [53]
Heifangtai	0.4288	0.0763	0.0519	2.1740	Cao C.W. [54], Gao Z.A. [55], Liu P.F. [56]
Tongchuan	0.5570	0.1740	0.0481	1.1700	Li P. [22]
Dongxiang	0.4275	0.1252	0.0628	2.1300	Liu P.F. [56]
Heyang	0.5620	0.1680	0.0521	1.2600	Li P. [22]
Zhengning	0.3926	0.0922	0.0595	2.0805	Li P. [57, 58], Zhang Y.Q. [59], Sun F. [60]
Baishui	0.5310	0.1870	0.0400	1.3200	Li P. [22]
Ansai	0.3613	0.0613	0.0534	1.7971	Zhang X. [61]
Zichang	0.4492	0.0678	0.0582	1.8746	Sun F. [60], Wang X.F. [62]
Baoji	0.2600	0.1220	0.0330	1.5730	Sun F. [60], Guo H. [63, 64]
Qishan	0.3975	0.1400	0.0416	1.0900	Guo H. [63, 64], Li P. [22]
Yanglin	0.2550	0.1200	0.0448	--	Guo H. [63, 64]
Xi’an	0.4243	0.0869	0.0813	1.5984	Fan S. [38], Wang T.X. [65], Yang T. [66], Han J.M. [67], Yue L.B. [68], Qiu H.B. [69]
Sanmenxia	0.2311	0.0537	--	--	Wang Y. [70]

**Note**: For multiple sets of parameters obtained in the same area, the final value selected is each Arithmetic average of parameters. For multiple values in the same area, use the 4σ criterion to eliminate outliers, and then calculate and use the average of the remaining values

**Fig 2:**
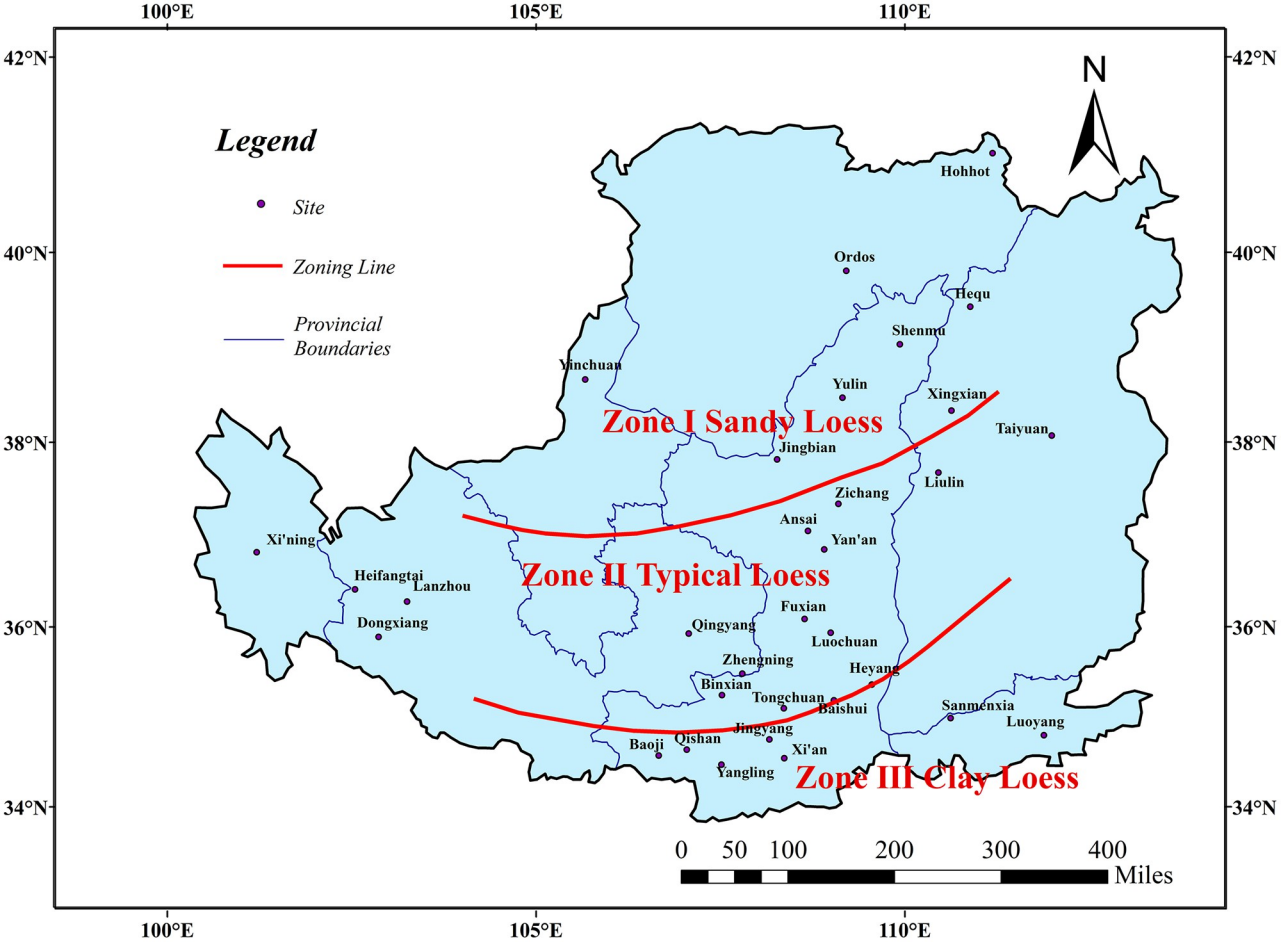
The zoning of the Chinese Loess Plateau(Only the boundary of the Loess Plateau comes from Huang [32], illustrative purposes only).

The sampling ranges are all located in the Malan loess stratum at a distance of 2-5m from the upper strata boundary, according to the soil properties and particle gradation, Fan et al. [38] divided the loess of the Loess Plateau into three zones, which are shown in Fig 2. By sorting out the sampling results, get the difference in particle size composition of each zone, which are shown in Table 2. The sandy loess (Zone I), has the highest sand content, clay content is 6%~11%, powder content is 55%~65% and sand content is 25%~33%. The typical loess (Zone II), contains a large amount of powder and sand and a small amount of clay, clay content is 7%~12%, powder content is 60%~70% and sand content is 20%~27%. The clay loess (Zone III), has the highest clay content and a large amount of powder, clay content is 10%~20%, powder content is 70%~75%, and sand content is 5%~14%. In this way, the collected data on VG model parameters of each zone can be statistically analyzed, which are shown in Table 3.

**Table 2:** Particle gradation components of selected the sampling results.

Zone	clay content	powder content	sand content
Zone I	6%~11%	55%~65%	25%~33%
Zone II	7%~12%	60%~70%	20%~27%
Zone III	10%~20%	70%~75%	5%~14%

**Table 3:** VG model parameters table for each zone.

Zone	*θ*_*s*_	*θ*_*r*_	*α*	*n*
Zone I	$\frac{0.2424-0.499}{0.3721}$	$\frac{0.0450-0.1418}{0.0795}$	$\frac{0.0120-0.0860}{0.0465}$	$\frac{1.3565-1.9320}{1.6931}$
Zone II	$\frac{0.3271-0.5620}{0.4561}$	$\frac{0.0368-0.1895}{0.1261}$	$\frac{0.0276-0.2002}{0.0678}$	$\frac{1.0200-2.1740}{1.5977}$
Zone III	$\frac{0.2311-0.4595}{0.3379}$	$\frac{0.0537-0.1400}{0.0973}$	$\frac{0.0330-0.1067}{0.0615}$	$\frac{1.0900-1.7005}{1.4905}$

Note: The data format is $\frac{min-max}{mean}$

According to the data in the Table 3, the parameters show obvious regularity. The average value of Parameter *θ*_*s*_ and *θ*_*r*_ in Zone II are both larger than the other two zones. The average value of Parameter *α* in Zone I is less than that in Zone III and Zone II. The average value of Parameter *n* is biggest in Zone I, second in Zone II and smallest in Zone III.

### Methods

#### Trend surface analysis method

Trend surface analysis is a multivariate statistical analysis method. It takes the geological variable Z as the dependent variable and the two-dimensional plane coordinates X and Y as the independent variables. A multi-form (high-order) regression equation can be constructed to form a plane or more complex spatial surface in geometry to reflect the geological variable in space. Therefore, it separates the regional variation characteristics (trends) of environmental variables in space from the local variation characteristics (abnormalities). It can also remove the regional variation component and highlight the local variation component, to study the abnormal points and analyze their causes [71, 72].

#### Radial basis function method

Radial basis function (RBF) is a real-valued function whose value depends only on the distance from the origin [73], that is

$$\Phi \left(x\right)=\varphi \left(\left\|x\right\|\right)$$ (2)

Or it can be the distance to any point c, where c is called the center point, that is

$$\Phi \left(x\right)=\varphi \left(\left\|x-c\right\|\right)$$ (3)

Any function *Φ*(*x*) that satisfies Eq (3) is called a radial basis function, and the distance is calculated by Euclidean distance [74, 75].

The radial basis function is an effective method to deal with the multivariate function approximation problem. The main work of its studies is the expansion of space surface and its properties, and applies this space surface to handle the description problem of the data. It has the advantages of high efficiency, simple operation, easy programming, convenient storage in the computer, isotropy, independent of grids, and high solution accuracy. It has been extensively researched and applied in many fields, such as, function approximation, neural networks, multi-scale analysis, mining, geophysics, surveying and mapping, remote sensing and signal processing and machine learning [76].

#### Universal Kriging

The variability of regional variables is represented by three parts: the deterministic part, the relevant part and the random part. The ordinary Kriging method requires variables to satisfy the second-order stationary assumption or inherent assumption, that is, assuming that the deterministic part is constant in space. For non-stationary variables, the deterministic part is not constant in space. It must be assumed that the deterministic part is distributed with space, also known as drift or tendency. The best linear estimation process corresponding to this method is called universal Kriging method [77–80].

Because the mean value is no longer a constant in space but a spatial variable, considering a regional variable *z(x)* defined in the study area *A* at position *x*, it can be represented by a deterministic drift *m(x)* and a residual part *r(x)*:

$$z\left(x\right)=m\left(x\right)+r\left(x\right)$$ (4)

Matrix representation:

Z=M+R (5)

Through the definition of drift, the expectation of *z(x)* at *x* is *m(x)*, that is

$$E\left[z\left(x\right)\right]=m\left(x\right),\;E\left[r\left(x\right)\right]=0$$ (6)

If the drift function is known, the drift can be minus in the original data. Then, if the remained residual part *r(x)* satisfies the inherent assumption, the residual part can be estimated by Kriging method. Hence, the estimated value can be obtained by plus the drift to the estimated residual part at relative position [79].

Assuming that *m(x)* can be expressed by a linear combination of functions *P*_*k*_ (*x*)(*k* = 1,2,…,*K*):

$$m\left(x\right)=\sum_{i=1}^{K}a_{k}p_{k}\left(x\right)$$ (7)

Matrix representation:

M=PA (8)

Where *a*_*k*_ is an unknown coefficient; *p*_*k*_ (*x*) is a known function of *x*, which is often expressed by a polynomial of *x*^*k*−1^.

$$m\left(a,n\right)=b_{0}a+b_{1}n+b_{2}a^{2}+b_{3}n^{2}+b_{4}an+b_{5}$$ (9)

Where **P** is (*n* × *k*) polynomial matrix; n is the number of data point; *p* is the number of drifts, which can be determined by multiple stepwise regression; **A** is the polynomial coefficient of drift; **R** is the theoretical residue.

Assuming that the residual part *r*(*x*) satisfies the second-order equilibrium condition, *σ*(*h*) is used to represent its covariance coefficient, the covariance of *r*(*x*′) and *r*(*x*) are only a function of distance *xx*′:

$$E\left[r\left(x\right)r\left(x^{\prime }\right)\right]=\sigma \left(xx^{\prime }\right)$$ (10)

Matrix representation:

$$E\left[RR^{\prime }\right]=S$$ (11)

Assume that *σ*(*h*) is known. The main problem of universal kriging is to determine the best coefficient *a*_*k*_ of the drift part [79].

#### Operation on ArcGIS

Importing the sorted data into ArcGIS for analysis, the radial basis function method is used for the measured value contour graph, because it has high efficiency when dealing with multiple problems, can handle large-scale scattered data, and has good approximation capabilities. The universal Kriging method is employed for the prediction value contour graph and the prediction standard error value graph, because it is a geo-statistical method that takes into account the unbiased linear estimator with drift. It can allow the existence of measurement errors.

By using ArcGIS for analysis, we can establish a more realistic way of predicting SWCCs and unsaturated permeability coefficients that can be updated at any time in the Chinese Loess Plateau. Because each piece of data is obtained from actual experiments, it is real and effective, and it is closer to the actual soil properties of the Chinese Loess Plateau. As the sampling density increases, its accuracy will gradually become more accurate.

Through the method below, we can analyze each VG model parameter of the Malan loess SWCCs in Chinese Loess Plateau.

Step I, input the map. Import study area boundaries(the boundary map of the Loess Plateau) and administrative division boundaries into ArcGIS as vector files. Superimposed these two vector files to obtain the administrative division map of the Loess Plateau in Arcmap.(Only the boundary of the Loess Plateau in this article comes from Huang [32] as a basemap for vectorization, illustrative purposes only).
Step II, import the Data. Add the geographic location points on the administrative division map of the Loess Plateau, and add the data of *α*, *n*, *θ*_*s*_ and *θ*_*r*_ to its attribute table.
Step III, perform the spatial deterministic interpolation. Utilize ***Radial Basis Function Interpolation Method*** function in ***Interpolation Analysis*** under the ***Geostatistical Analyst Tool*** in ***Arctoolbox***. After inputting the data of *α*, *n*, *θ*_*s*_ and *θ*_*r*_ respectively, employ the boundary layer of the Loess Plateau in ***environment*** as the processing range, so as to obtain the measured value contour graph of each parameter of the VG model.
Step IV, perform the geostatistical interpolation. Utilize the ***Geostatistical Wizard*** command under ***Geostatistical Analyst*** in the toolbar. Select the ***Kriging/CoKriging interpolation method*** in the pop-up dialog box, and enter the *α*, *n*, *θ*_*s*_ and *θ*_*r*_ data respectively. Then, choose ***universal kriging method*** in the ***kriging method type list box***, employ ***prediction*** and ***prediction standard error*** respectively in the output type list box, and then config the required factor to generate the prediction value contour graph and the prediction standard error value graph for each parameter value of the VG model.
Step V, perform the trend surface analysis. Utilize ***Trend analysis*** in ***Explore data*** under ***Geostatistical Analyst*** in the toolbar, and input *α*, *n*, *θ*_*s*_ and *θ*_*r*_ data to obtain the trend analysis graph of each parameter.

## Results

### VG model parameter *α*

**Fig 3:**
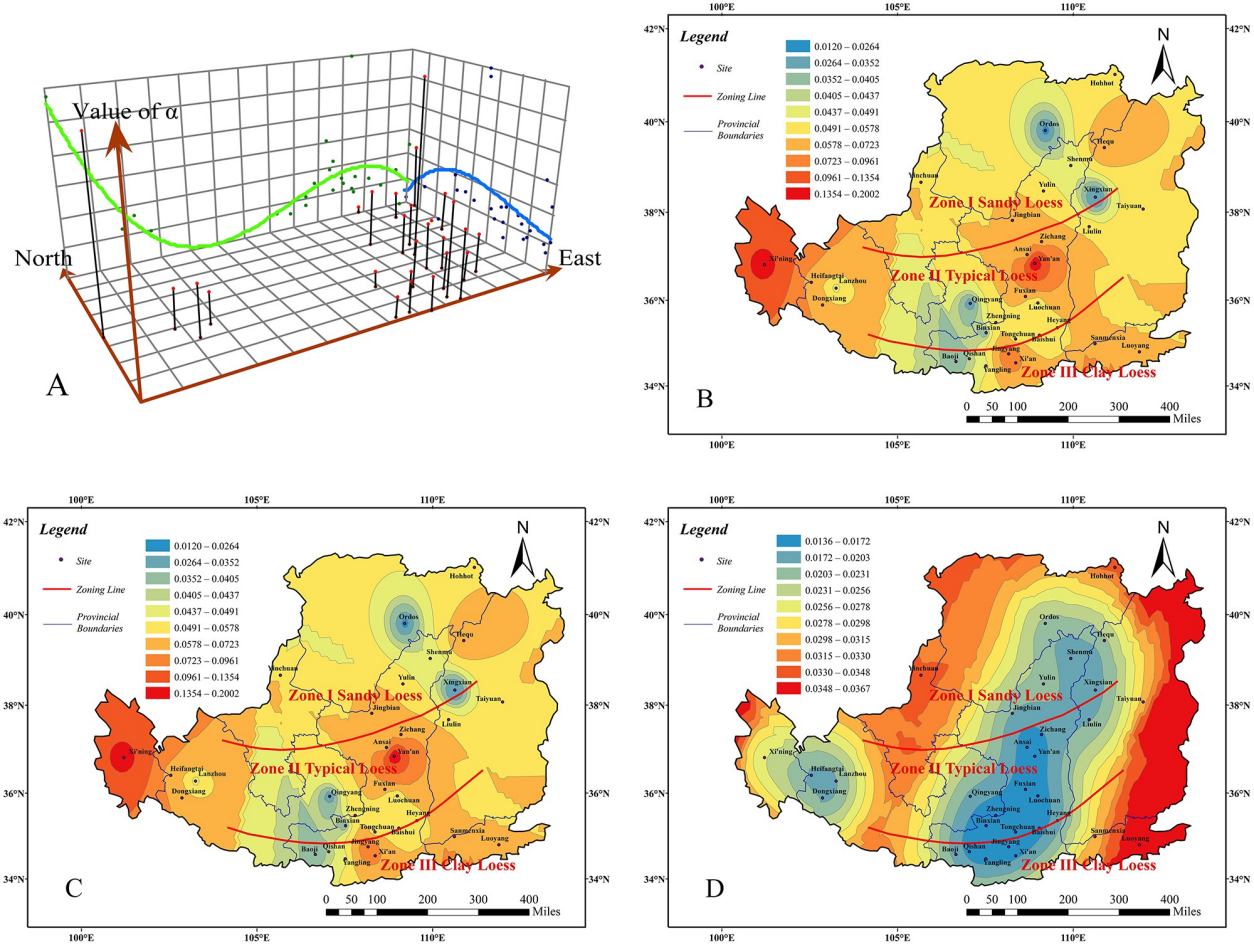
Analysis result of parameter *α*. Trend analysis graph(A). measured value contour graph(B). prediction value contour graph(C). prediction standard error value graph(D) (Only the boundary of the Loess Plateau comes from Huang [32], illustrative purposes only).

From the trend analysis graph (Fig 3A) and the prediction value contour graph (Fig 3C), the trend regular pattern of the VG model parameter α in the Chinese Loess Plateau can be obtained. The measured value contour graph (Fig 3B) can illustrate the spatial distribution of the VG model parameter α. The prediction standard error value graph (Fig 3D) reflects the accuracy of the predicted value of the VG model parameter α and the abnormal situation of the data. Combining these four graphs, the regional distribution of VG model parameter α in the Chinese Loess Plateau is obtained as follows:

1. The trend value of the parameter *α* (A) is higher in the southeast and lower in the northwest. It decreases from the west to the east and then increases gradually. Overall, the value in the west is higher than in the east.
2. The measured value of parameter *α* (B) is basically higher in Zone II, followed by Zone III, and lower in Zone I. Among them, the values are the higher (0.135~0.2) at Zichang of Shaanxi province and Xining of Qinghai province in Zone II, it is the lowest (0.012~0.02) at Ordos in the Chinese Loess Plateau.
3. The predicted value of parameter *α* (C) is higher in Zone II and Zone III, and lower in Zone I. Among them, Ansai, Yan’an and Zichang in north-central Shaanxi province, Sanmenxia in northern Henan province and Xining in Qinghai province have higher values (0.07~0.09). Southern Ningxia province, eastern Gansu province and Ordos have the lowest values (0.01~0.02).
4. The prediction standard error value of the parameter *α* (D) has little deviation between the measured value and the predicted value in most areas, especially in the central and southern parts of Shaanxi province in the Chinese Loess Plateau, where the error values are generally less than 0.017. However, in the Luoyang area, the error value is larger (0.03). But most error values are small, which certify the predicted value are accurate in most areas.

### VG model parameter *n*

**Fig 4:**
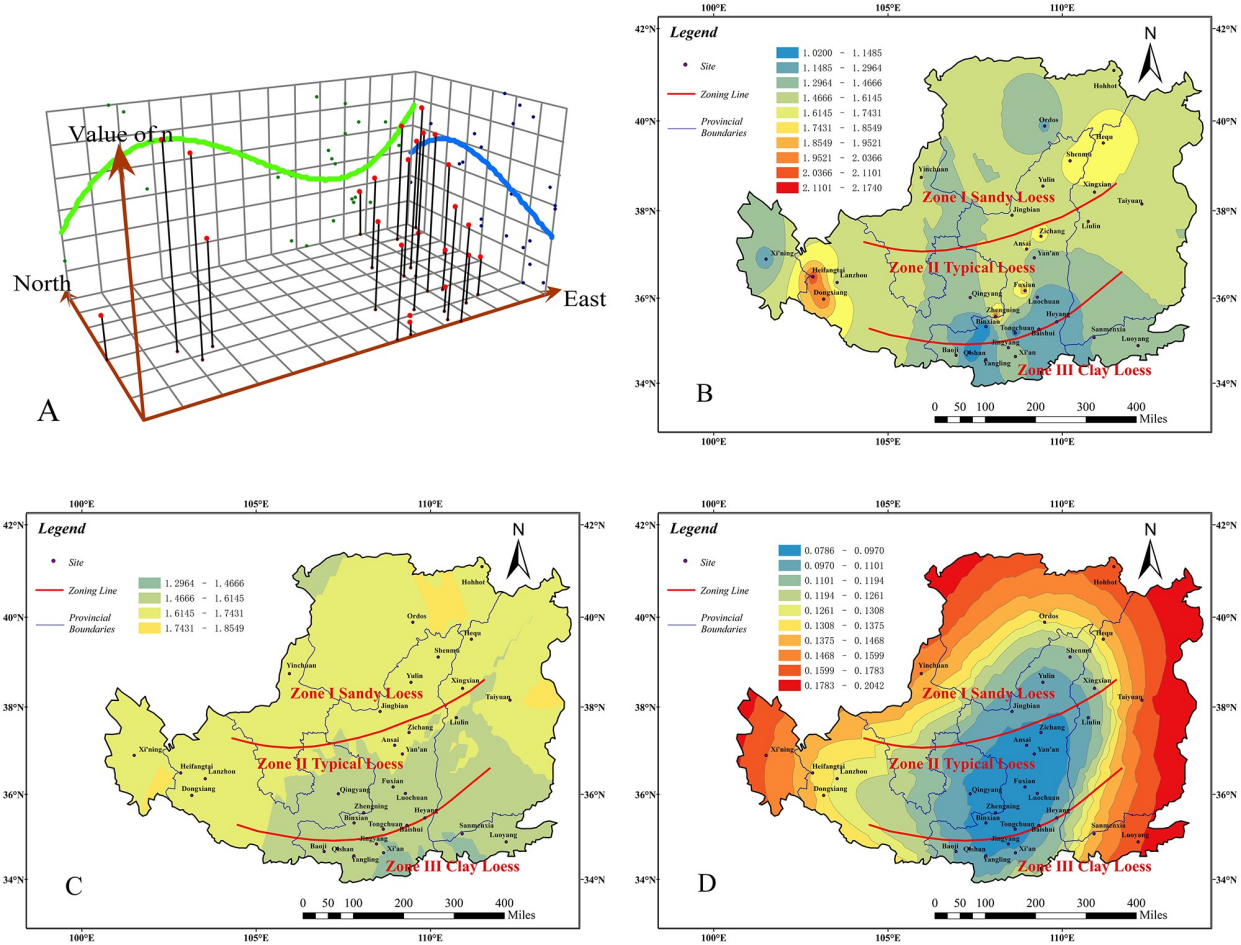
Analysis result of parameter *n*. Trend analysis graph(A). measured value contour graph(B). prediction value contour graph(C). prediction standard error value graph(D) (Only the boundary of the Loess Plateau comes from Huang [32], illustrative purposes only).

By the same analysis method, we can acquire the regional distribution of VG model parameter n in the Chinese Loess Plateau, which is shown as follows:

1. From the trend analysis graph (Fig 4A) and the prediction value contour graph (Fig 4C), we can see that the trend value of the parameter *n* is higher in the north and lower in the south. It decreases from the east to the west and then increases gradually. Overall, it is higher in the northwest than in the southeast.
2. As shown in Fig 4B, the VG model parameter *n* is higher obviously in Zone I, followed by Zone II, and lower in Zone III. Among them, the values are higher (2.03~2.11) at Heifangtai and Dongxiang in Gansu province than other areas in Zone II. And Qishan is the lowest (1.02~1.14), which is in Zone III.
3. From Fig 4C, we can see that the predicted value of parameter *n* is highest in Zone I, second in Zone II, and lowest in Zone III, and its value varies significantly with the three divided zones. The highest value is in the eastern part of Ordos (1.74~1.85), and the lowest values (1.29~1.46) are in the eastern part of Xi’an and the southern part of Sanmenxia.
4. As described in Fig 4D, the prediction standard error value of the parameter *n* reflects a little deviation between the measured value and the predicted value in the majority of areas, especially in the central Shaanxi province and eastern Gansu province, where the error values are generally less than 0.09. However, in Xining of Qinghai province, the error value is the biggest (0.15~0.17). The error values are small and attest predicted value of parameter n are accurate in most areas.

### VG model parameter *θ*_*s*_

**Fig 5:**
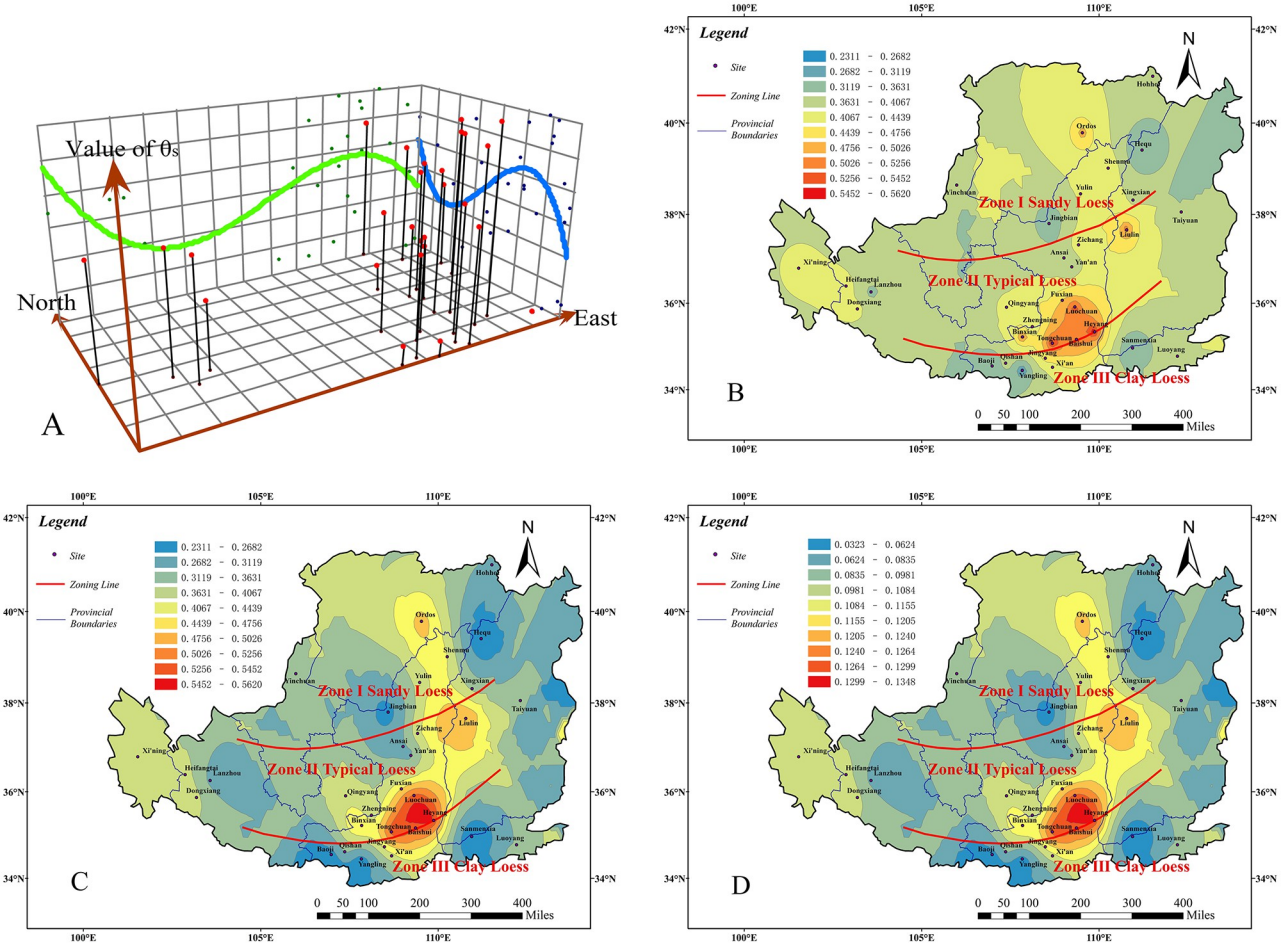
Analysis result of parameter *θ*_*s*_. Trend analysis graph(A). measured value contour graph(B). prediction value contour graph(C). prediction standard error value graph(D) (Only the boundary of the Loess Plateau comes from Huang [32], illustrative purposes only).

From these four graphs, the regional distribution of VG model parameter *θ*_*s*_ in the Chinese Loess Plateau can be obtained as follows:

1. As shown in Fig 5A and 5C, the trend of the VG model parameter *θ*_*s*_ is higher in the southeast and lower in the northwest. It decreases from the west to the east and then increases gradually. Overall, it is higher in the east than in the west. And it increases slowly after gradually decreasing from the north to the south. Overall, it is higher in the south than in the north.
2. The measured value contour graph (Fig 5B) can illustrate that the VG model parameter *θ*_*s*_ is higher in Zone II obviously, which the high value is relatively centralized. Then, Zone III is the second, and lower in Zone I. Among them, they are the higher value (0.52~0.56) in the triangle area of Tongchuan, Heyang and Luochuan in Shaanxi province, which is in Zone II. Liulin in Shanxi province and Ordos in Inner Mongolia province are followed (0.47~0.5). In the entire Chinese Loess Plateau, it is the lowest (0.23~0.31) in Yangling, Jingbian in Shaanxi province and Hequ in Shanxi province. In northern Ningxia province and western Inner Mongolia province (0.31~0.4) were lower too.
3. Fig 5C shows that the predicted value of parameter *θ*_*s*_ is higher in Zone II, and lower in Zone I and III. Among them, Luochuan, Tongchuan, and Heyang in Central Shaanxi, Liulin in Western Shanxi, and Ordos in Inner Mongolia this band shape area have higher values. Among them, Luochuan, Tongchuan, and Heyang in central Shaanxi have the highest values in the triangle area (0.52~0.56). Other regions, such as Northwestern Shanxi, Southern part of Yangling, and Northern part of Jingbian to Western Inner Mongolia have the lowest values (0.23~0.31).
4. The prediction standard error value graph (Fig 5D) reflects that the accuracy of the predicted value of the VG model parameter *θ*_*s*_ has a little deviation between the measured value and the predicted value in most areas, especially in most areas of Shaanxi province, western Shanxi province, and the belt shape region from Xining to Lanzhou, the error values are generally less than 0.06. However, in Yinchuan of Ningxia province and Hohhot of Inner Mongolia province, the error values are larger (0.124~0.129).

### VG model parameter *θ*_*r*_

**Fig 6:**
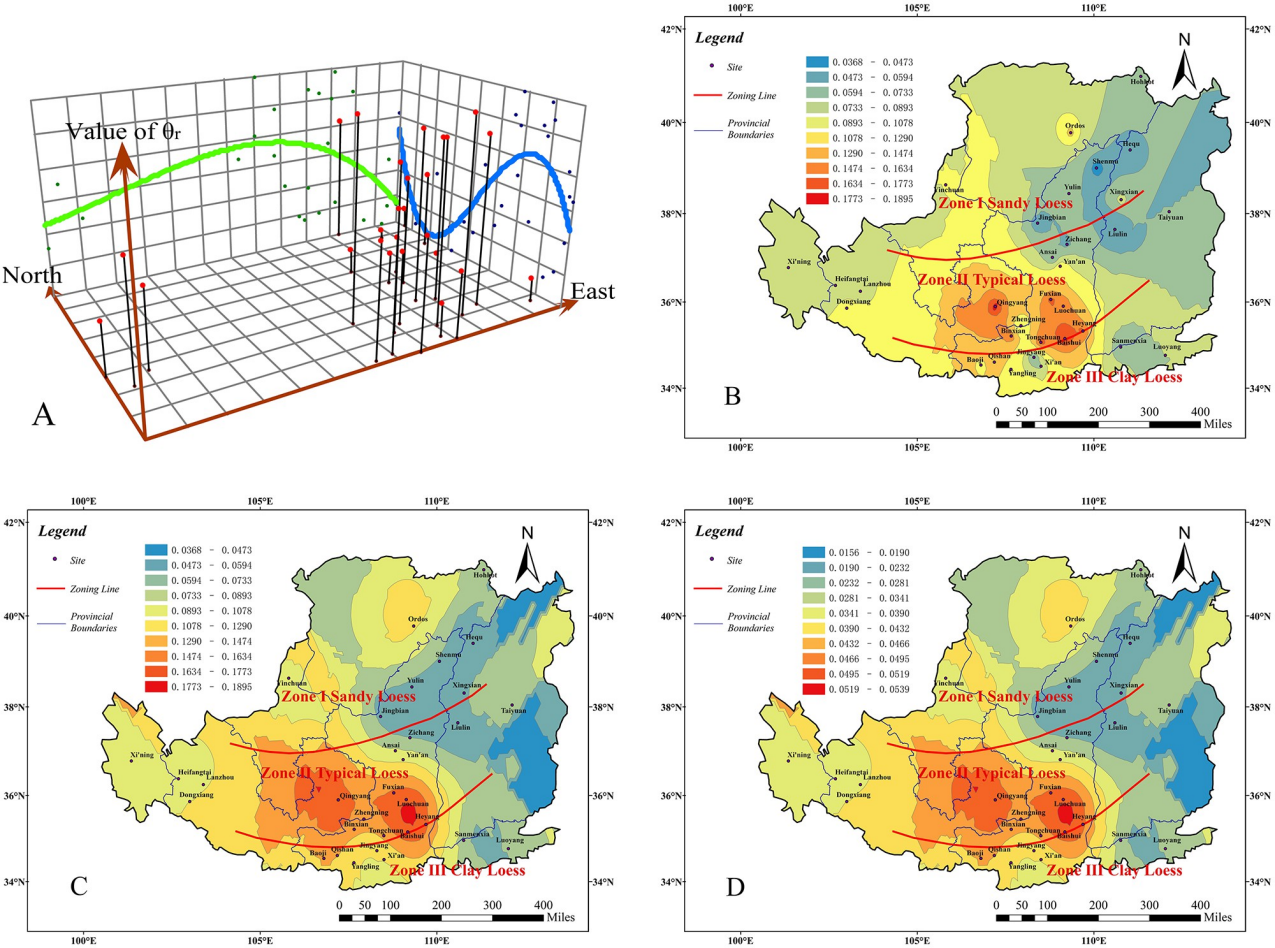
Analysis result of parameter *θ*_*r*_. Trend analysis graph(A). measured value contour graph(B). prediction value contour graph(C). prediction standard error value graph(D) (Only the boundary of the Loess Plateau comes from Huang [32], illustrative purposes only).

From Fig 6, we can get the regional distribution of VG model parameter *θ*_*r*_ in the Chinese Loess Plateau, which is shown as follows:

1. From Fig 6A and 6C, the trend regular pattern of the VG model parameter *θ*_*r*_ in the Chinese Loess Plateau can be obtained. It is higher in the southeast and lower in the northwest. It rises gradually from west to east and then falls rapidly. Overall, it is higher in the east than in the west. In the north-south direction, it descends rapidly from north to south and then rises slowly. Overall, the trend value in the south is higher than in the north.
2. Fig 6B can illustrate the spatial distribution of the VG model parameter *θ*_*r*_. It is higher in Zone II obviously, followed by Zone III, and lower in Zone I. Among them, Baishui of Shaanxi province and Qingyang of Gansu province have the highest values (0.16~0.18), which is in Zone II. While the values in northern Shaanxi province and northwest Shanxi province are lower. The Shenmu in the north of Shaanxi province is the lowest (0.03~0.04).
3. From Fig 6C, we can see that the predicted value of parameter *θ*_*r*_ is higher in Zone II and Zone III, and lower in Zone I. Among them, Jingbian, Yulin, and Shenmu in north-central Shaanxi province, Hequ and Xingxian in northwest Shanxi province have lower values (0.03~0.04). The highest values are in eastern Gansu province and southern-central Shaanxi province (0.17~0.18).
4. Fig 6D reflects that the accuracy of the predicted value of the VG model parameter *θ*_*r*_ has little deviation in southern-central of Shaanxi province and Lanzhou and it is generally less than 0.019. In Xining of Qinghai province, Yinchuan of Ningxia province and Hohhot of Inner Mongolia province, the error values are larger (0.05). The error value in Shaanxi Province is relatively small, less than 0.03.

## Discussion

### Analysis on the reasons of distribution

Combined with scholars’ research on SWCC and the prediction map, the property of SWCCs on the Loess Plateau can be analyzed as follows.

The previous work on unsaturated soil has indicated that the particle size composition of loess has an important influence on the SWCCs of loess. If the content of gross particles is high, the pores of the loess are larger and it is easier to form a drainage channel [20]. In addition, the specific surface area of loess is small, so the adsorption capacity of water will be reduced. Therefore, the air intake value of gross particle soil is relatively small, the dehumidification rate is relatively large, and the residual moisture content is relatively low [38, 81]. If the content of fine grains of loess are high and the size of soil particles are small, the pores of the loess are small and the drainage is more difficult [20]. Because of the large specific surface area of the loess, the adsorption capacity for water is strong. Therefore, the adsorption capacity of clay particles is the strongest, powder particles is second, and the sand particles is the weakest. At same time, the higher the fine particle composition is, the greater the air intake value is, the smaller the dehumidification rate is, and the greater the residual moisture content is [38, 81].

For the parameter *α*, studies have shown that it has a higher correlation with the air entry value. The larger the air entry value is, the smaller the parameter α is [81–83]. It also shows that the higher the fine particle content of loess is, the greater the value of the parameter α is. As the content of fine particles is relatively high in the central and southern parts of the Chinese Loess Plateau, while the northern area is mainly sandy loess, hence, the α values of Zone II and Zone III are higher than those in Zone I. The reason that some areas have the abnormal value is that the air entry value is not only affected by the particle composition, but also controlled by the loess porosity [38], and its value is also susceptible to external disturbances during the measurement process.

For the parameter *n*, studies have shown that it is a parameter related to the moisture reduction rate. If the moisture reduction rate is high, the value of parameter *n* is large too. [81–83]. That is, the higher the fine particle content of the loess is, the smaller the value of the parameter *n* should be. Because the clay loess area and the typical loess area have more fine particles, and the sand loess area has more gross particles, the value of parameter *n* is low in Zone II and III and high in Zone I. And, therefore, the prediction map of the parameter *n* shows that it’s low in the southeast and high in the northwest consistently.

For the parameter *θ*_*s*_, its value is mainly related to the porosity ratio of loess. The higher the porosity is, the greater the saturated water content of the loess has. Because of the fine particle content of loess, the void ratio is great. It can be inferred that the tinier the loess particles is, the larger the parameter *θ*_*s*_ should be. According to the analysis of the particle size division of the Chinese Loess Plateau and the spatial trend of the parameter *θ*_*s*_, it can be concluded that the parameter *θ*_*s*_ is higher in the southeast and lower in the northwest, and the high values are basically distributed on both banks of the Yellow River. It is presumed to be a large amount of fine particle matter brought by the alluvium of river. Therefore, the high value of the parameter *θ*_*s*_ gradually increases along the direction of the Yellow River.

The higher the content of fine particles is, the stronger the adsorption capacity to water is, and the greater the value of the parameter *θ*_*r*_ is [20, 81]. Combining the particle size division of the Chinese Loess Plateau and the spatial trend analysis of the parameter *θ*_*r*_, it can be concluded that the loess in the southern contains more clay and powder particles, and the northern part contains more sand particles. Therefore, the parameter *θ*_*r*_ has a larger value in the southern, and it has smaller value in the northern of Chinese Loess Plateau. It is basically consistent with the predicted map of the parameter *θ*_*r*_.

### Calculation method of unsaturated permeability coefficient

By applying the hydraulic conductivity Eq (12) [18] and combining with the statistical trend data to deduce the unsaturated permeability coefficient of a certain place.

$$k=k_{s}S_{e}^{1/2}{\left[1-{\left(1-S_{e}^{1/m}\right)}^{m}\right]}^{2}$$ (12)

Where k is the unsaturated permeability coefficient; *k*_*s*_ is the saturated permeability coefficient; *S*_*e*_ is the effective saturation, it can be calculated by Eq (13).

$$S_{e}=\frac{1}{{\left[1+{\left(\alpha \psi \right)}^{n}\right]}^{m}}$$ (13)

Where, ψ is the matrix suction, *kPa*; α and n are VG model fitting parameters.

It can be seen from the formula that only *α*, *n*, *ψ* and *k*_*s*_ are required to derive the unsaturated permeability coefficient *k*.

**Fig 7:**
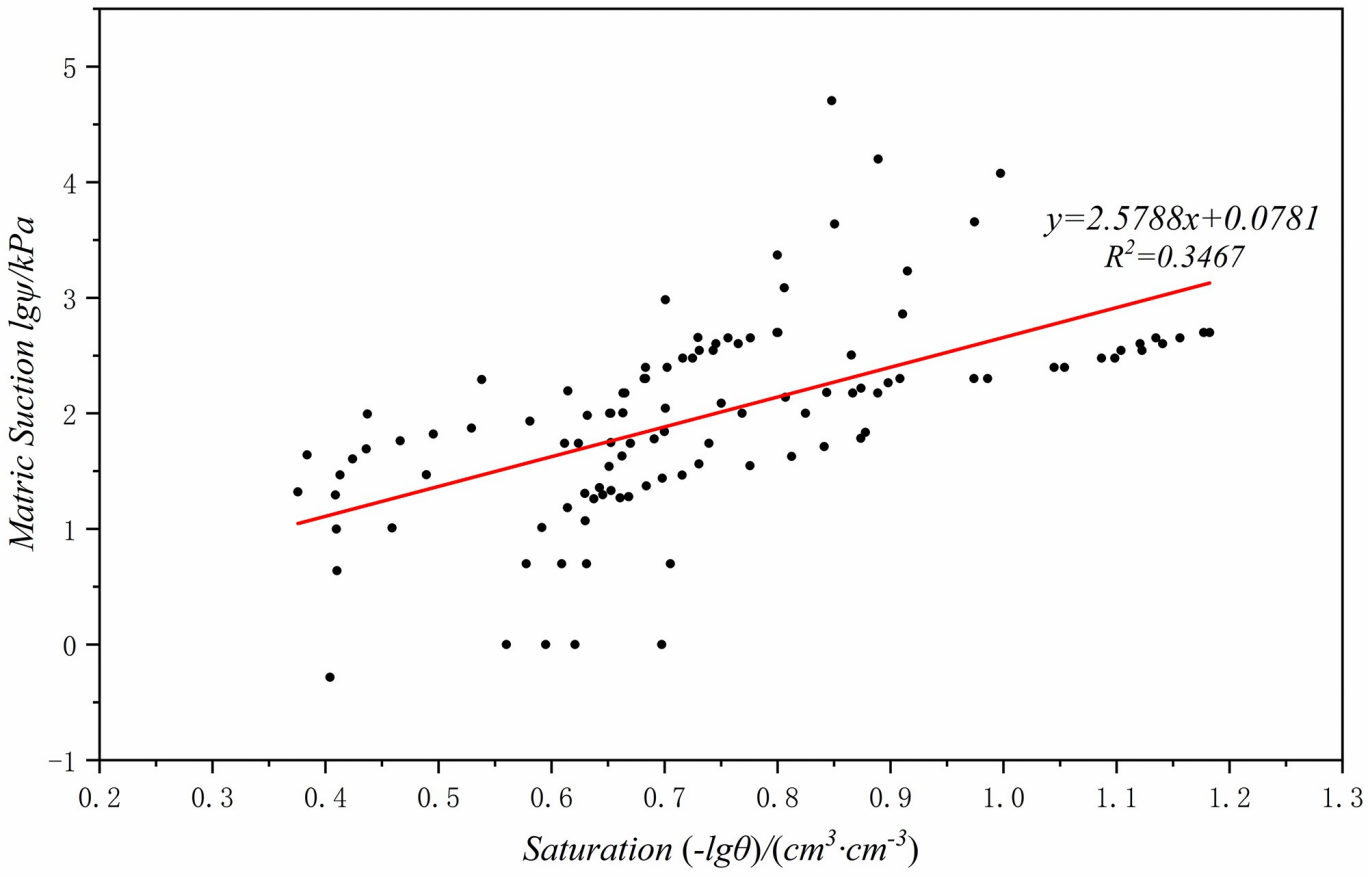
Sandy loess (Zone I) degree of saturation and matric suction fitting result.

**Fig 8:**
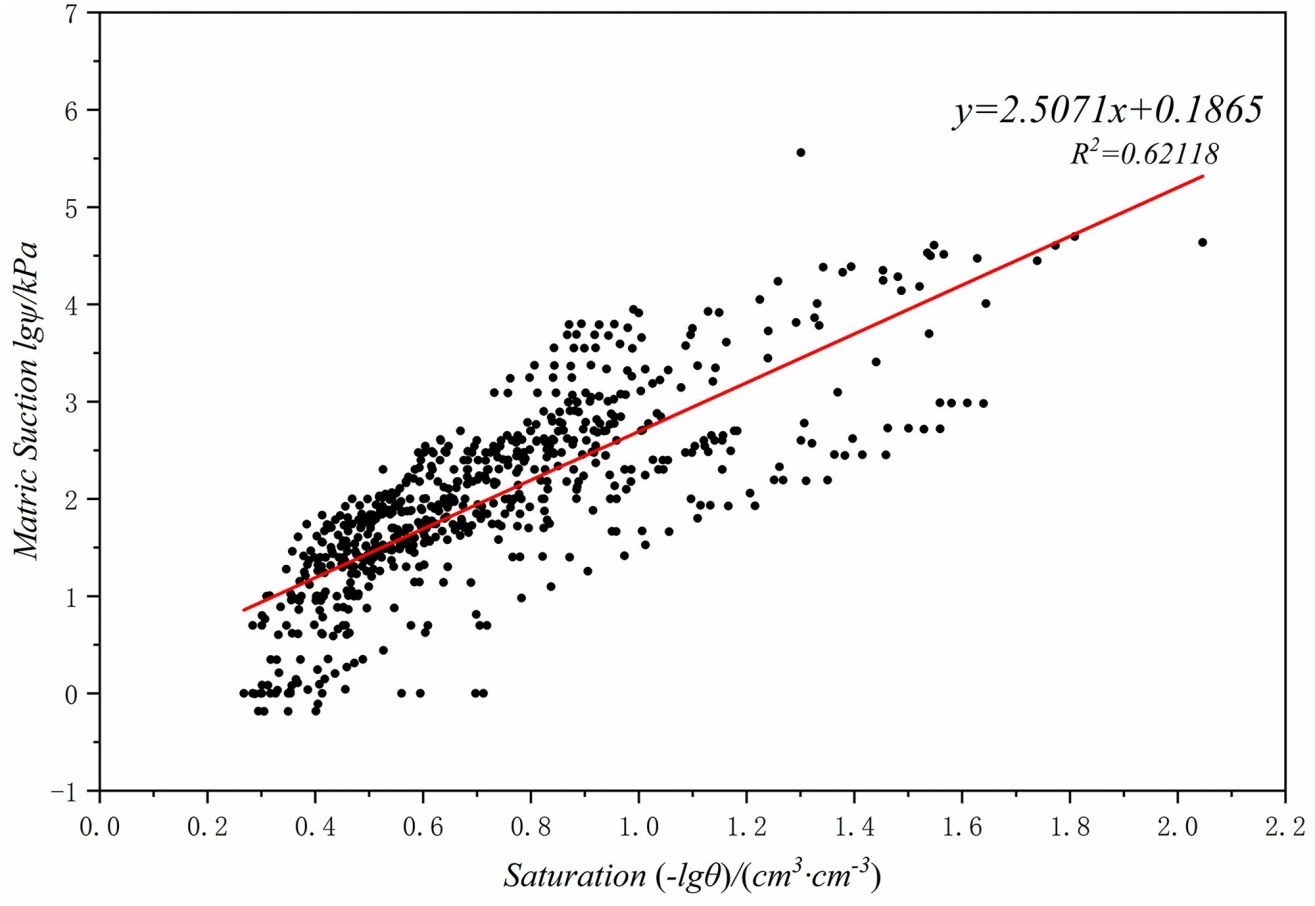
Typical loess (Zone II) degree of saturation and matric suction fitting result.

**Fig 9:**
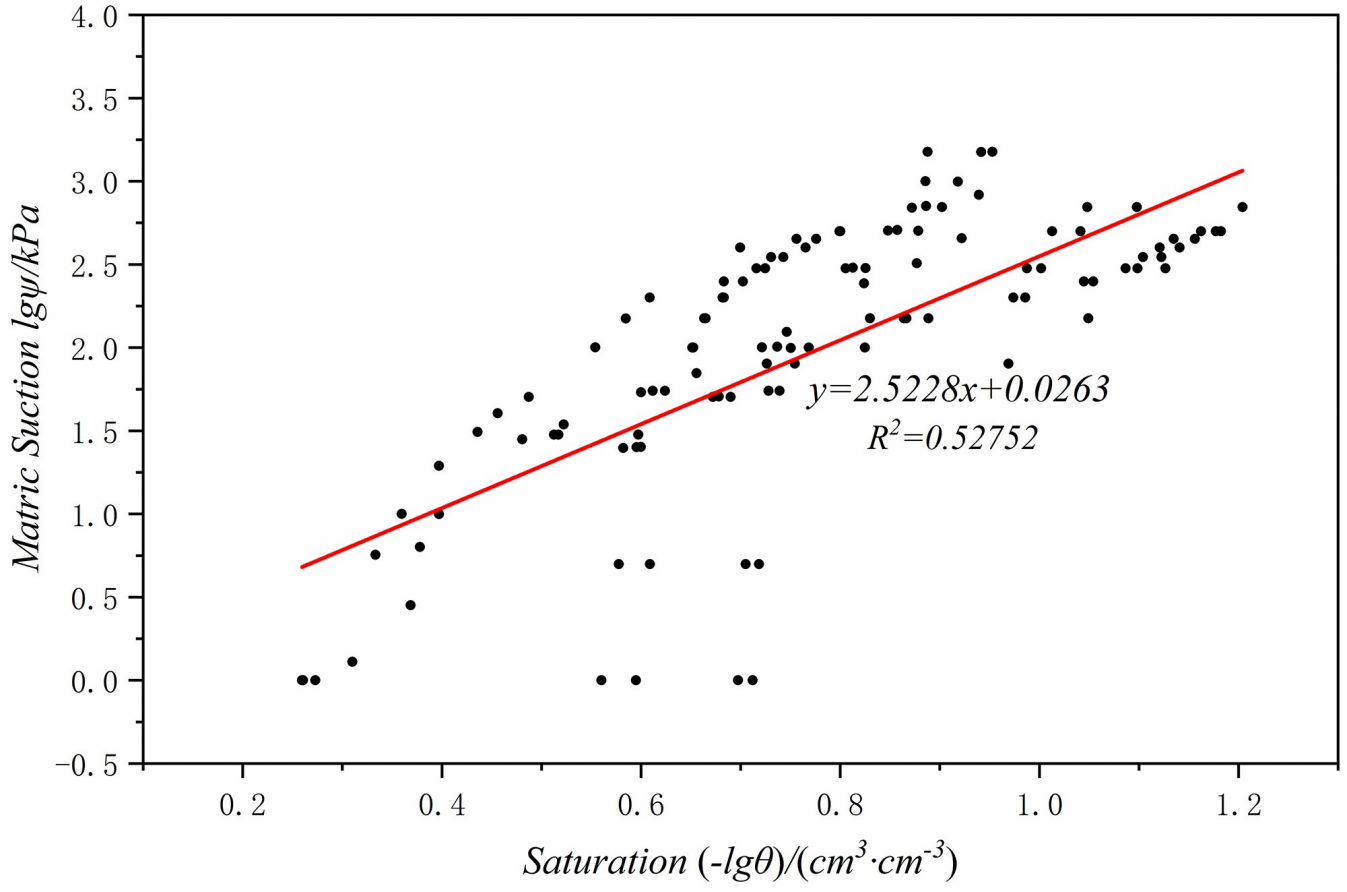
Clay loess(Zone III) degree of saturation and matric suction fitting result.

Import the matrix suction and saturation data that have been tested in the Loess Plateau region into Origin for data transformation, draw a scatter plot (Figs 7–9), and then perform linear fitting to obtain the fitting formulas between the matrix suction and saturation in three different regions of Chinese Loess Plateau (14–16).

lgψ=−2.5788lgθ+0.0781 (14)

lgψ=−2.5071lgθ+0.1865 (15)

lgψ=−2.5228lgθ+0.0263 (16)

Where, *ψ* is the matrix suction, *kPa*; *θ* is the volumetric moisture content, *cm*^*3*^*/cm*^*3*^.

If you want to obtain the unsaturated permeability coefficient of a certain sample in the Loess Plateau, read the value of *α*, *n*, *θ*_*s*_, *θ*_*r*_ at the certain place on the prediction map firstly. Then, select one formula (14–16) according to the certain place in which zone in Chinese Loess Plateau and calculate the matrix suction *ψ*. Thirdly, calculate m through n and substitute it into the formula (13) to obtain the effective saturation *S*_*e*_. After measuring the saturated permeability coefficient *k*_*s*_ of the sample and using the formula (12), we can gain the unsaturated permeability coefficient *k*.

This method is simple and economical, compared with fractal method and artificial intelligence algorithm, this method has a wider application range and can reflect the changes of parameters in the region. And it saves a lot of computational troubles (for example, the fractal method needs to analyze the more troublesome pore and particle characteristics), and the data can be obtained quickly. It can provide a preliminary guidance for engineering construction on a large scale. But due to the various measurement methods of the sampled data and different experimental standards, the loess samples will be disturbed to different degrees, so some values will have a certain degree of error. For the parameter α that is greatly affected by the experiment and the void ratio, it is recommended to use the formula for calculating the dry density of each area to assist in the calculation of the parameter α in the actual application. For example, Ji [84] gave the formula for estimating the parameter *α* of dry density in the Heifangtai area, Luo [85] gave the formula for estimating the parameter *α* of dry density in Binxian, Shaanxi, and Zhu [86], Ren [87], similar research has also been done. In the actual situation, we can refer to more literature and choose the best. If the testing data of VG model parameters in more and more areas we have, the prediction accuracy of the unsaturated permeability coefficient will be further improved. In order to solve the engineering problems related to loess and water better, and give the better guidance on engineering construction, future research should further focus on the prediction accuracy.

### Calculation example verification

The measured results of other scholars’ unsaturated permeability coefficients in different zones are used to verify the prediction accuracy of this method, which is shown in Table 4.

**Table 4:** Unsaturated permeability coefficient data source.

Data source	area	Zone
Hu H.J. [88]	Yangling	Zone III
Wang H.M. [89]	Yan’an	Zone II
Yang Y. [90]	Jingbian	Zone I

The value ranges of the VG model parameters, obtained by reading the contour graph in different zones, are shown in Table 5.

**Table 5:** The prediction data of VG model parameter by contour graph.

	Yangling		Yan’an		Jingbian	
	min	max	min	max	min	max
***α***	0.0491	0.0579	0.0723	0.0962	0.0579	0.0722
***n***	1.4667	1.6145	1.6145	1.7431	1.6145	1.743
***θ***_***s***_	0.2311	0.2683	0.3119	0.3631	0.2311	0.2683
***θ***_***r***_	0.1290	0.1474	0.1078	0.1290	0.1290	0.1474

**Fig 10:**
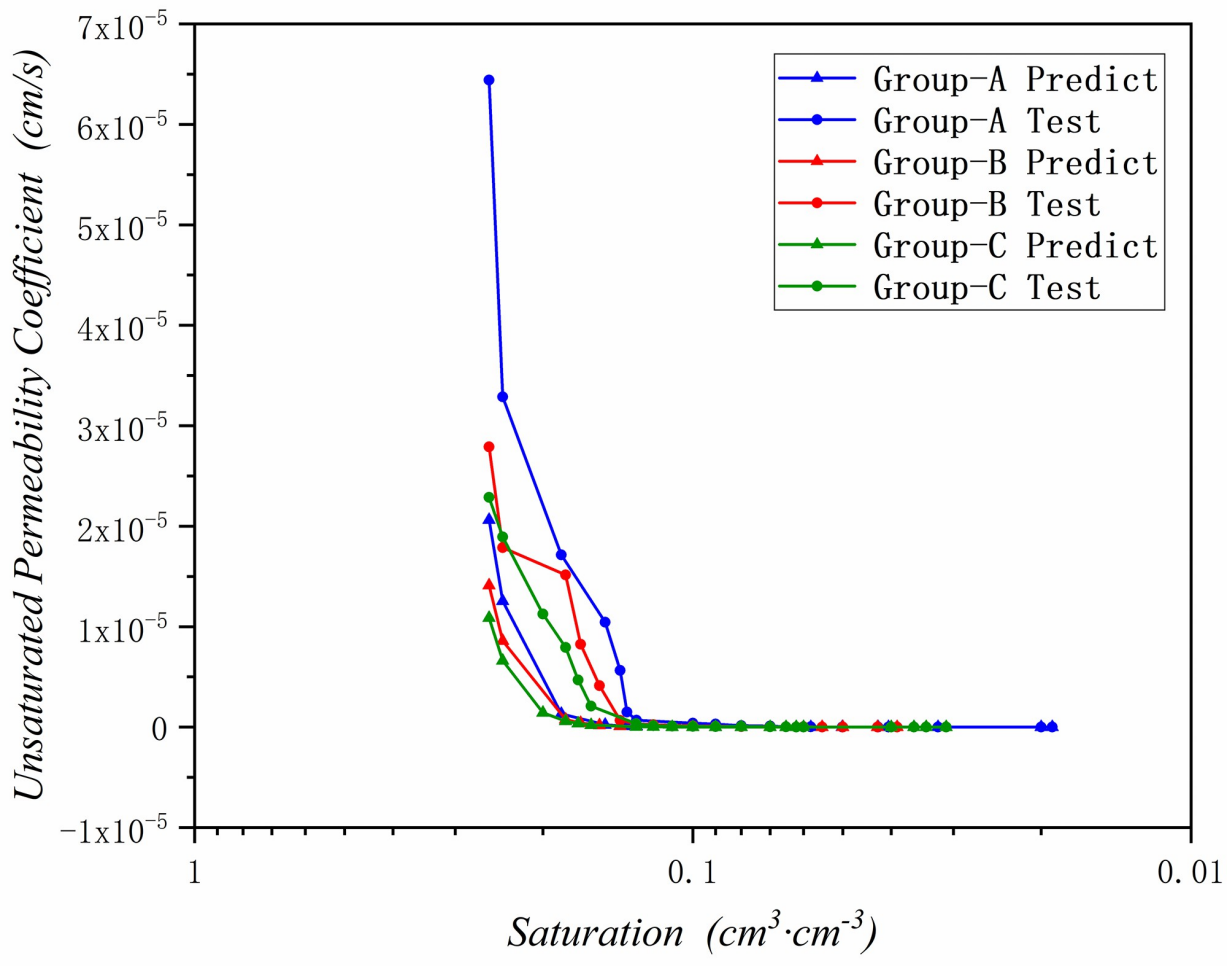
The predicted unsaturated permeability coefficient graph in Zone III(Yangling).

**Fig 11:**
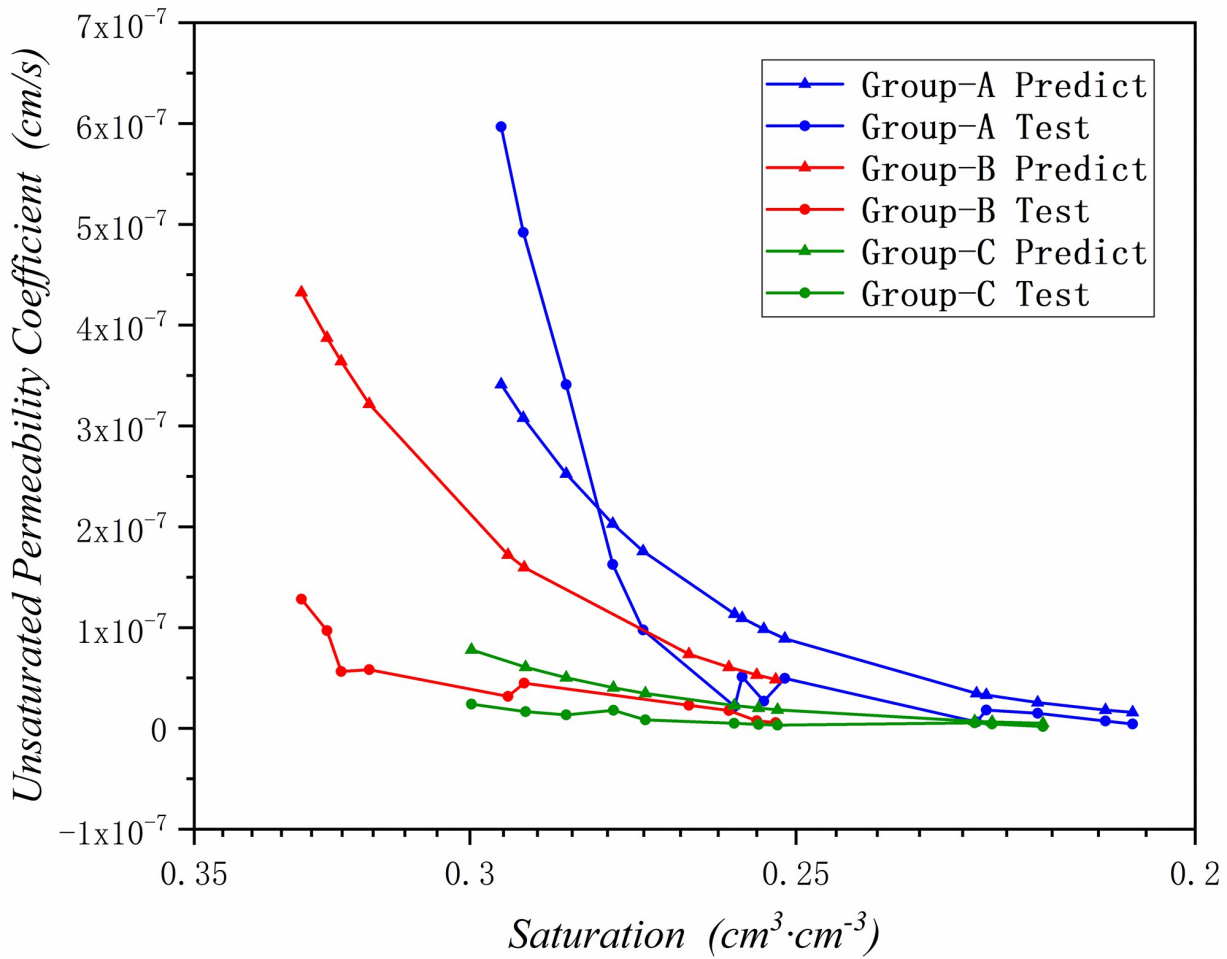
The predicted unsaturated permeability coefficient graph in Zone II(Yan’an).

**Fig 12:**
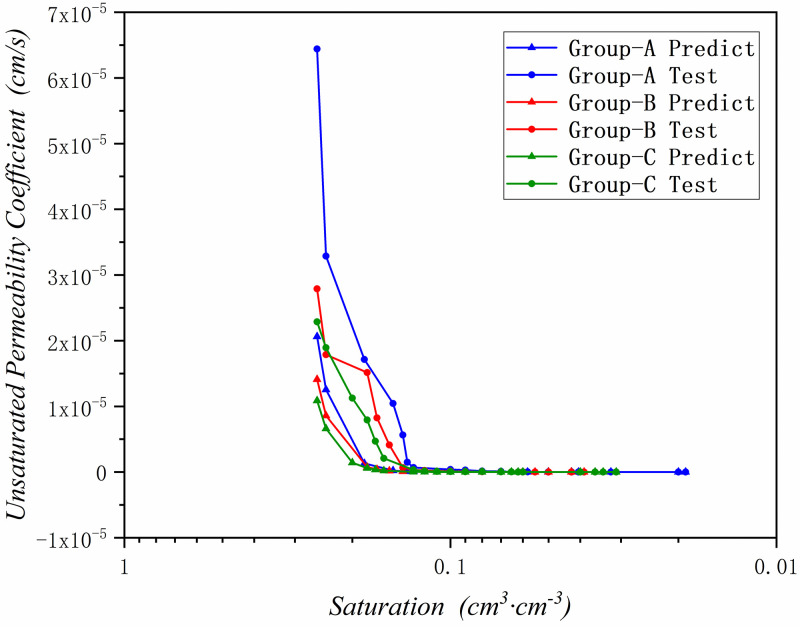
The predicted unsaturated permeability coefficient graph in Zone I(Jingbian).

By using the proposed method, the unsaturated permeability coefficient has been predicted in these three research areas. And compared the predict results with the experimental test data, the results are been shown in Figs 10–12 and Table 6.

**Table 6:** The prediction deviation of the prediction results.

Zone	Group	Deviation		
		Max	Min	Average
Zone I	A	*4*.*38e*^*-5*^	*1*.*94e*^*-10*^	*5*.*19e*^*-6*^
	B	*1*.*44e*^*-5*^	*1*.*93e*^*-10*^	*3*.*16e*^*-6*^
	C	*1*.*23e*^*-5*^	*1*.*94e*^*-10*^	*2*.*56e*^*-6*^
Zone II	A	*2*.*56e*^*-7*^	*1*.*06e*^*-8*^	*5*.*19e*^*-9*^
	B	*3*.*08e*^*-7*^	*4*.*27e*^*-8*^	*1*.*60e*^*-7*^
	C	*5*.*37e*^*-8*^	*1*.*41e*^*-9*^	*2*.*17e*^*-8*^
Zone III	A	*2*.*29e*^*-6*^	*1*.*25e*^*-8*^	*5*.*08e*^*-7*^
	B	*3*.*72e*^*-6*^	*4*.*41e*^*-8*^	*8*.*03e*^*-7*^
	C	*1*.*44e*^*-6*^	*2*.*22e*^*-8*^	*4*.*77e*^*-7*^

According to Figs 10–12 and Table 6, it can be seen that the prediction results are basically accurate. The three test groups in the Zone III have the same saturated permeability coefficient, so they only have one predicted result.

It can be found in Fig 10 and Table 6 that the prediction results are basically in line with the experimental results, and the prediction deviation is basically controlled within *±4*.*77e*^*- 7*^ *cm*/*s*. When the saturation of soil sample is low, the deviation is small, and it is basically controlled at *±2*.*22e*^*-8*^*cm*/*s*. When the saturation of soil sample is high, the unsaturated permeability coefficient is large, and the prediction deviation is also large at lower saturation stage, but it is also controlled at *±2*.*29e*^*-6*^*cm*/*s*. And the transition between the predicted result and the saturated permeability coefficient is relatively smooth, which is more in line with the actual situation.

In Fig 11 and Table 6, comparing the prediction results with the experimental test results, the prediction results are basically accurate, and the prediction deviation is basically controlled within *±2*.*17e*^*-8*^*cm*/*s*. The predicted results in the lower saturation section of soil sample are in good agreement with the experimental results, with a deviation of *±1*.*06e*^*-8*^*cm*/*s*. When the saturation of soil sample is higher, the unsaturated permeability coefficient becomes larger, and the prediction deviation is larger than soil sample in lower saturation stage, which is basically controlled at *±3*.*08e*^*-7*^ *cm*/*s*, and it can be seen that in the experimental test results, some unsaturated permeability coefficient rebounded. The results of this situation are generally caused by experimental deviations, and the prediction results can make up the test deviation.

In Fig 12 and Table 6, since the unsaturated permeability coefficient of sandy loess in Zone I is the largest, the difference between the predicted results and the experimental results is basically *±3*.*16e*^*-6cm/s*^. However, the prediction results are extremely accurate at low saturation of soil sample, and the deviation is basically *±1*.*94e*^*-10*^*cm*/*s*. In the higher saturation section of soil sample, the prediction deviation is also larger due to the higher saturation and the influence of the higher permeability coefficient of sandy loess, with a deviation of *±1*.*44e*^*-5*^*cm*/*s*. For the larger unsaturated permeability coefficient in the sandy loess area, the deviation is within the acceptable range.

By predicting the unsaturated permeability coefficients of the three regions, it can be concluded that: for approximately estimating the unsaturated permeability coefficient of a certain place, the unsaturated permeability coefficient of a certain place in the Loess Plateau can be obtained quickly and economically only by a relatively simple experiment proposed in this paper. The prediction result of the method of permeability coefficient is relatively accurate, the deviation is very small in the lower saturation section, and the deviation is slightly larger in the higher saturation section, but it is also within the acceptable range.

## Conclusion

The SWCCs reflects the relationship between water content and matrix suction. It has been widely used to estimate the loess properties. The unsaturated permeability coefficient of loess is the key to solving loess engineering problems. Therefore, research on the SWCCs fitting model is very important. In view of this, the VG model parameters of the SWCCs data in the Loess Plateau region studied by experts and scholars in recent years are collected in this paper, and trend analysis and one-variable linear regression methods are used to study the regional distribution law of the VG model parameter and the calculated method on unsaturated permeability coefficient is developed. The conclusions are shown as the following.

1. The parameters of the VG model in the Loess Plateau have obvious regularities:
  ①For the parameter *α*, the value is high in the area with high fine particle content, such as the southeast of the Chinese Loess Plateau, and it is low in the area with high gross particle content, like, in the northwest. There are abnormal values in some areas such as Zichang of Shaanxi province and Xining of Qinghai province in Zone II.
  ②For the parameter *n*, the value is low in the areas with high fine particle content, for example, in the southeast, and it is high in the areas with high gross particle content, like in the northwest.
  ③For the parameter *θ*_*s*_, it is high in the southeast and low in the northwest, and the high value is basically distributed on both banks of the Yellow River. It is speculated that a large amount of fine particle matter is brought about by the alluvium of the river.
  ④For the parameter *θ*_*r*_, the value is large in the south and small in the north. And this trend is mainly related to the average particle size of loess.
2. Through linear fitting of matrix suction and saturation, the linear formulas of them are obtained in three different zones on the Chinese Loess Plateau. As long as the *k*_*s*_ of a loess sample in a certain area are obtained, the unsaturated permeability coefficient of the sample can be derived by reading the *α*, *n*, *θ*_*s*_, *θ*_*r*_ of the certain place on the prediction map and using these fitting formulas. The prediction result of the method of permeability coefficient is relatively accurate, the deviation is very small in the lower saturation section, and the deviation is slightly larger in the higher saturation section, but it is also within the acceptable range. This method can greatly reduce the experiment cost. It will provide convenience for solving the engineering problems related to loess and water and other engineering applications.

## Supporting information

S1 Table The data summary of Van Genuchten model parameters on soil-water characteristic curves in Chinese Loess Plateau. (XLS)
